# Population Genomics of Commercial Fish *Sebastes schlegelii* of the Bohai and Yellow Seas (China) Using a Large SNP Panel from GBS

**DOI:** 10.3390/genes15050534

**Published:** 2024-04-24

**Authors:** Beiyan Zhu, Tianxiang Gao, Yan He, Yinquan Qu, Xiumei Zhang

**Affiliations:** 1 Fishery College, Zhejiang Ocean University, Zhoushan 316022, China; ziky9911@163.com (B.Z.); gaotianxiang0611@163.com (T.G.); xiumei1227@163.com (X.Z.); 2 MOE Key Laboratory of Marine Genetics and Breeding, College of Marine Life Sciences, Ocean University of China, Qingdao 266003, China; yanhe@ouc.edu.cn

**Keywords:** *Sebastes schlegelii*, population genomics, GBS, SNPs, genetic differentiation, genetic diversity

## Abstract

*Sebastes schlegelii* is one of the most commercially important marine fish in the northwestern Pacific. However, little information about the genome-wide genetic characteristics is available for *S. schlegelii* individuals from the Bohai and Yellow Seas. In this study, a total of 157,778, 174,480, and 188,756 single-nucleotide polymorphisms from Dalian (DL), Yantai (YT), and Qingdao (QD) coastal waters of China were, respectively, identified. Sixty samples (twenty samples per population) were clustered together, indicating shallow structures and close relationships with each other. The observed heterozygosity, expected heterozygosity, polymorphism information content, and nucleotide diversity ranged from 0.14316 to 0.17684, from 0.14035 to 0.17145, from 0.20672 to 0.24678, and from 7.63 × 10^−6^ to 8.77 × 10^−6^, respectively, indicating the slight difference in genetic diversity among *S. schlegelii* populations, and their general genetic diversity was lower compared to other marine fishes. The population divergence showed relatively low levels (from 0.01356 to 0.01678) between *S. schlegelii* populations. Dispersing along drifting seaweeds, as well as the ocean current that flows along the western and northern coasts of the Yellow Sea and southward along the eastern coast of China might be the major reasons for the weak genetic differentiation. These results form the basis of the population genetic characteristics of *S. schlegelii* based on GBS (Genotyping by Sequencing). In addition to basic population genetic information, our results provid a theoretical basis for further studies aimed at protecting and utilizing *S. schlegelii* resources.

## 1. Introduction

*S. schlegelii* belongs to the genus *Neohispaniscus* (Scorpaenidae), widely distributed in the northwestern Pacific [1]. In China, *S. schlegelii* mainly inhabits the Bohai Sea to the Yellow Sea and the East Sea of China, usually spending the majority of its life amid reef-associated water, gravel, and caves [2]. *S. schlegelii* is an economical fish species with delicious and nutritious flesh, which is widely appreciated by consumers in China, Republic of Korea, and Japan [3], but it also has an ecological value as a demersal carnivorous fish [4]. Adults and those in the early-life stages constitute an important link in the transfer of energy and matter within the pelagic and benthic trophic webs as well as in the benthopelagic coupling of the region [5]. Due to the advantages of fast growth, a low temperature tolerance, and high stress resistance, *S. schlegelii* is an important marine culture and commercial fishing fish in the northwestern Pacific [6]. The aquaculture production of *S. schlegelii* can reach up to 33,020 metric tons per year [7].

Given the increasingly prominent issues of overfishing and changes in the environment of inshore fishing grounds, the fishery resources of *S. schlegelii* have been gradually decreased, and the genetic diversity of wild populations has been seriously affected [8]. There are important economic and social benefits to breeding high-quality varieties with fast growth rates and high disease resistance from wild populations for artificial breeding. For instance, the quantity of wild fishery resources can be increased to a certain extent by adding artificially bred seedlings upon the transfer to natural waters for stock enhancement [9], so as to restore fishery resources, protect biological diversity, maintain ecological balance, and promote the sustainable development of fishery [10]. Hence, stock enhancement has become an important measure for the recovery and management of offshore fishery resources in China [11]. However, the inflow of artificially bred seedlings may affect the genetic structure, diversity, and divergence of the original wild population, and unscientific breeding may also have a negative impact on the wild resource population [12].

For the process of artificial breeding with seedlings of marine fishes, one of the key issues is to ensure the genetic diversity of parents or seedlings for stock enhancement. Therefore, molecular biotechnology has been widely used to ensure the genetic population structure, diversity, and divergence of the wild populations [13,14,15]. Previous studies have explored the genetic diversity and structure of *S. schlegelii* among different populations (Bohai Sea, Yellow Sea, and Japan Sea) based on various genetic markers. For instance, Zhang et al., found no significant population genetic structure of *S. schlegelii* corresponding to sampling locations in China, Japan, and Republic of Korea using a mitochondrial DNA sequence (mtDNA) control region [16,17]. Another two investigations using microsatellites and amplified fragment length polymorphism (AFLP) markers both showed no significant genetic differentiation among populations of *S. schlegelii* from China and Japan, indicating a single gene pool among populations [8,18]. To date, the population structure and genetic divergence based on genome-wide markers of the *S. schlegelii* species remain poorly reported. In our study, we sequenced the genomes of *S. schlegelii*, including 60 accessions from three coastal waters (Yantai, Dalian, and Qingdao) of China. Population structure, genetic diversity, and genetic divergence of the *S. schlegelii* populations were investigated. Candidate genomic loci or genes involved in some important functions, such as the regulation of DNA recombination, ribosomal large subunit assembly, and mitochondrial genome maintenance, have been identified in *S. schlegelii* through genomic selective sweep analyses. The single-nucleotide polymorphism (SNP) panel and derived genomic analyses could serve as a reference for fishery resource protection, improved species breeding, and the stock enhancement of *S. schlegelii*.

## 2. Materials and Methods

### 2.1. Sample Collection and DNA Extraction

**Figure 1:**
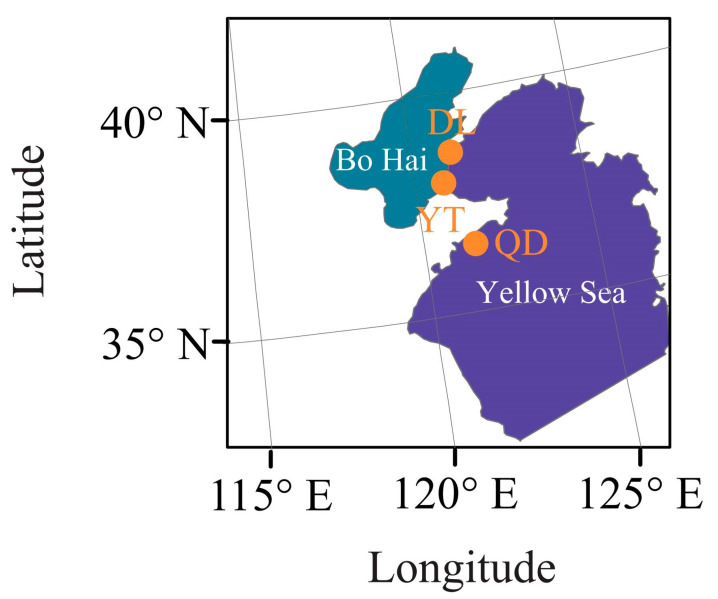
Collection locations of three populations of *S. schlegelii*.

To explore the population structure and genetic diversity of the *S. schlegelii* population, we sequenced 60 samples from 2 geographic areas distributed in the Yellow Sea (20 samples for Qingdao (QD) with sampling codes QD1 to QD20) and Bohai Sea (20 samples for Yantai (YT) with sampling codes YT1 to YT20, and Dalian (DL) with sampling codes DL1 to DL20, respectively) of the northwestern Pacific covering the DL (38°70′ N, 121°16′ E), YT (37°88′ N, 120°76′ E), and QD (36°21′ N, 120°77′ E) coastal waters of China (Figure 1). DL and YT coastal waters are located in the north temperate zone, with no extreme heat in summer and no severe cold in winter. The annual variation in water temperature in QD coastal waters is greater than that in YT and DL coastal waters, in which the climate is characterized by cold and dry winters and warm and humid summers. The sampling locations are in close proximity to each other (Figure 1). Fresh muscles on the right side of the back of the fish were preserved in liquid nitrogen and stored at −80 °C for further experiment. Total DNA was extracted from muscle tissues using a standard phenol–chloroform method [19]. The DNA concentration and purity (OD260/OD280 ratio) were determined by using a NanoDrop spectrophotometer 2000C (Thermo Scientific, Waltham, MA, USA), and DNA integrity was confirmed by 1.0% agarose gel electrophoresis. The qualified DNA precipitation was dissolved in sterilized ddH_2_O for further library construction [20].

### 2.2. GBS Library Construction, Illumina Sequencing, and Read Mapping

The restriction endonucleases HaeIII and EcoRI were used to digest the genome DNA. First, a P1 adapter, sequencing primer, and barcode were added to enzyme digestion products together with a ligase. Second, a P2 adapter was added to the sheared and pooled DNA fragments with a ligase. Third, the adaptor-ligated DNA sequences were amplified using polymerase chain reaction (PCR). The AxyPrep DNA gel extraction kit (AxyGEN, Union City, CA, USA) was selected to obtain the products with a 200 bp fragment size, and 1 ng/μL concentration was prepared for subsequent analyses. The insert sizes were detected by an Agilent 2100 Bioanalyzer system (Agilent, Santa Clara, CA, USA) and the effective concentration of the libraries was quantified by qPCR (effective concentration > 2 nm).

The required fragments (200–400 bp) were selected for library construction. All libraries were sequenced based on the Illumina (San Diego, CA, USA), HiseqTM2500 (2 × 150 bp read length). GBS libraries and the sequencing itself were carried out by Novogene Co., Ltd. (Beijing, China). Finally, raw data for each sample were generated, and the quality of raw reads was checked by FastQC v0.11.5 [21]. The poor-quality sequences (<average Q30), short sequences (<50 bp), primer sequences, and adapter sequences were removed and excluded from the raw reads.

High-quality sequencing data from each sample were aligned to a reference genome (the assembled genome can be obtained by assembly ID CNA0000824 [22]) by using BWA v0.7.17 (Burrows-Wheeler Aligner) software with the settings “mem-t 4-k 32-M” [23]. The alignment reads with Sequence Alignment/Map (SAM) format were extracted and converted into Binary Alignment/Map (BAM) format, and then sorted according to genomic positions by SAMtools v1.9 with a maximum of 1000 reads at a position per BAM file [24]. BAM files had PCR duplicates removed using Picard v2.24.0 (MarkDuplicates) with the settings “REMOVE_DUPLICATES = true”, and unmapped reads and secondary alignments were removed using SAMtools with the “-r” option. Additionally, a minimum mapping quality (Q) score threshold of “-q 30” was applied to filter out low-quality alignments in BAM files using SAMtools.

### 2.3. Variant Calling, Population Structure, Diversity, and Divergence

The variant calling was carried out by using GATK v.4.0.3.0 [25] following the best practices workflow [26]. The general variants were identified for each individual using GATK HaplotypeCaller, and then combined by GenotypeGVCFs function to a single variant calling file. This two-step approach was carried out to ensure variant accuracy, which included re-genotyping and quality recalibration in the combined vcf file. SNPs were then identified using samtools/bcftools with default parameters based on alignments of all Illumina short reads. SNPs were filtered according to the following parameters: (1) SNPs were only present in one of the two pipelines (SAMtools/BCFtools and GATK); (2) SNPs with a read depth more than 1000 or less than 5; (3) non-biallelic SNPs; (4) SNPs with a missing rate more than 40%; (5) SNPs in repeat regions; (6) SNPs were removed if the distance with nearby variant sites was less than 5 bp. 

A phylogenetic gene tree was constructed based on SNPs in the single-copy genes regions. Two popular programs (RAxML v8.2.11 [27] with the GTRCAT model and IQ-Tree v1.6.12 [28] with the self-estimated best substitution model) were applied to construct the maximum likelihood (ML) tree. The PCA (principal component analysis) was performed using GCTA software v1.94.1 [29]. The ancestral population structure among individuals from the Bohai and Yellow Seas was estimated from the number of ancestral population units (K = 2–4) by using ADMIXTURE software v.1.3.0. Admixture analysis was performed following the parameter standard errors, which estimated by bootstrapping (bootstrap = 200). To investigate the genetic diversity and divergence among the two geographical areas, we evaluated the observed heterozygosity (*Ho*), expected heterozygosity (*He*), polymorphism information content (*PIC*), nucleotide diversity (*π*), and population divergence (F*_ST_*) based on the filtered SNP information using the program Stacks v2 and PLINK v1.90 [30,31].

### 2.4. Selection Pressure Analysis

To identify selective sweeps, Tajima’s D (which accounts for allelic variation) with a 100 kbp sliding window on the larger SNP data set was estimated using VCFtools v0.1.17 [32]. Three populations (QD, DL, and YT) from two geographic areas (Bohai and Yellow Seas) were, respectively, selected for selective analysis. The gene ontology (GO) functional annotation analysis was performed using the clusterProfiler R package v3.14.0 [33] with pvalueCutoff = 0.05, pAdjustMethod = “BH”, and TERM2GENE = term2gene.

## 3. Results

### 3.1. Sequencing and Variants Calling

In this study, 12.81 Gb of clean data (1,841,342 reads, 87.12% of raw reads with 2,113,568 reads) was generated after strict filtration and normalization with an average of 213.48 Mb of clean data per sample. A total of 199,811, 222,675, and 239,152 variants were, respectively, identified from DL, YT, and QD populations, including 157,778, 174,480, and 188,756 SNPs, as well as 42,033, 48,195, and 50,396 InDels (Insert/Deletion). Among them, SNPs from 60 samples were employed for analyses of genetic diversity and divergence and genome-wide selection pressure. After filtration, 149,434 SNPs were applied to the population structure analyses of the three populations (QD, DL, and YT) (Table S1). The ratio of transition to transversion (T*_S_*/T*_V_*) in SNPs was 2.48. We also identified that the three populations contained 107,709, 120,457, and 129,432 SNPs in intergenic regions, 88,180, 98,059, and 105,455 in introns, and 4444, 4810, and 4948 SNPs in exons, including 2377, 2690, and 2662 being synonymous, respectively (Table 1). 

### 3.2. Population Structure of S. schlegelii

**Figure 2:**
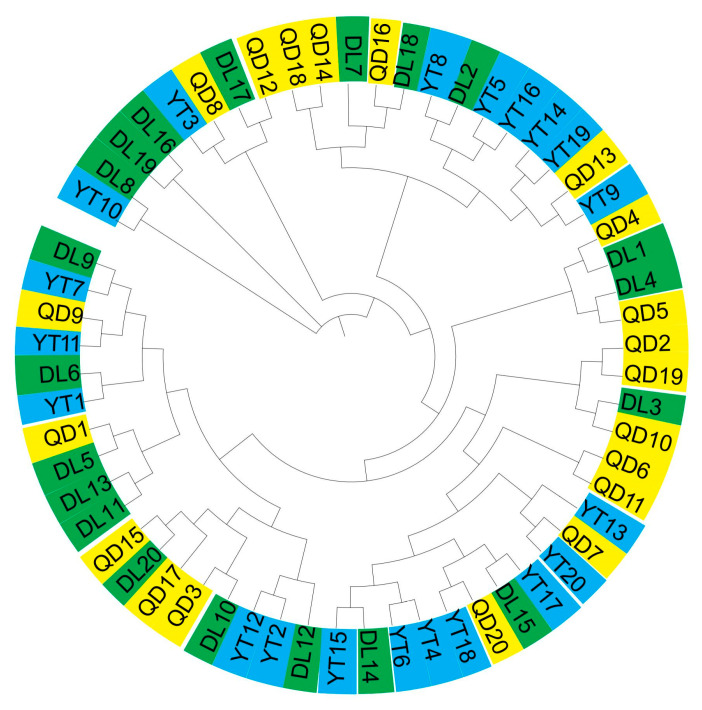
Phylogenetic relationship of 60 *S. schlegelii* individuals.

A phylogeny of these *S. schlegelii* individuals collected from two geographic areas was constructed (Figure 2). The 60 samples from different populations were clustered together, indicating shallow structures and close relationships with each other. The topology could not be generally defined by geographic localities; for instance, an amount of individuals from DL showed mixed components from QD and YT.

**Figure 3:**
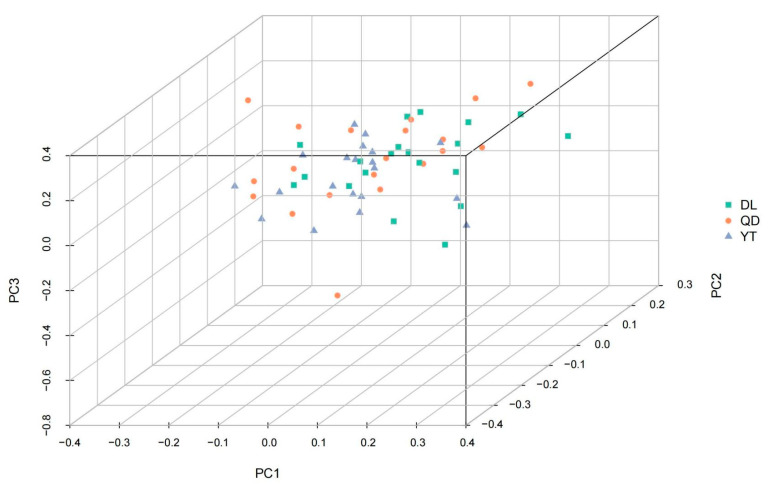
PCA analyses of the three *S. schlegelii* populations. Each sample is represented by a triangle.

PCA was applied to cluster individuals into different geographic populations according to their characteristics based on the SNP differences at the genome level (Figure 3). None of the three principal components (PC1, PC2, and PC3) were able to distinguish among the DL, YT, and QD populations. 

**Figure 4:**
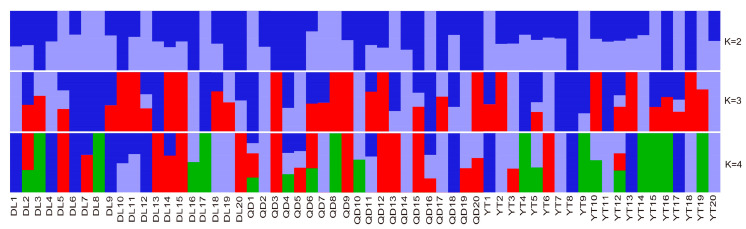
ADMIXTURE plot for *S*. *schlegelii* showing the distribution of K = 2, 3, and 4 genetic clusters among them. Each color represents a genetic cluster.

The population structure among two *S. schlegelii* geographical areas was estimated from population sizes K = 2–4 by using ADMIXTURE analyses (Figure 4). The population structure was determined and supported the phylogenetic topology and PCA analyses. The structure among DL, YT, and QD populations showed no obvious genetic differentiation among different geographical populations, indicating gene flow among these populations.

### 3.3. Genetic Diversity and Divergence of S. schlegelii Population

To investigate the genetic diversity and divergence among the two geographical areas (QD, DL, and YT), we observed a relatively high level of genetic diversity in the YT population (*π* = 8.77 × 10^−6^) relative to DL (*π* = 8.39 × 10^−6^) and QD (*π* = 7.63 × 10^−6^). The observed heterozygosity (*Ho*) measures the proportion of individuals in a population that are heterozygous at a given locus. In this study, the *Ho* for the DL, QD, and YT populations was 0.14316, 0.15809, and 0.17684, respectively (Table 2). The expected heterozygosity (*He*) for the DL, QD, and YT populations was 0.14035, 0.15420, and 0.17145, respectively (Table 2). The polymorphism information content (*PIC*) ranged from 0 to 0.5, with higher values indicating greater informativeness [34]. *PIC* values for the DL, QD, and YT populations were 0.20672, 0.22616, and 0.24678, respectively (Table 2), indicating that the SNP markers used in this study are moderately informative. Additionally, the differences in the three parameters (*Ho*, *He*, and *PIC*) among the three populations were not obvious, indicating that there was little variation in the level of observed heterozygosity between the populations. These results suggest that the populations have similar levels of genetic diversity.

**Figure 5:**
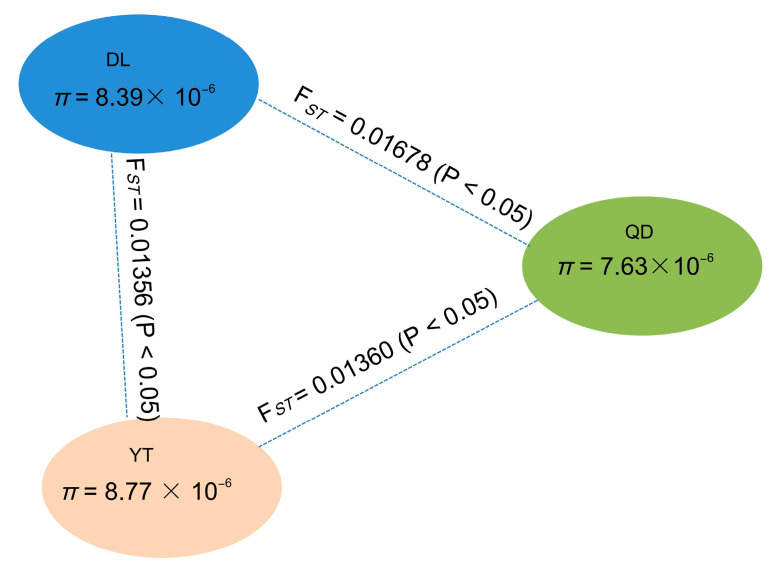
A summary of nucleotide diversity (*π*) and population divergence (F*_ST_*) across the three populations. The values in parentheses represent the measures of nucleotide diversity of each group, and the values between pairs indicate population divergence.

A low but significant genetic differentiation among all populations was detected based on Global F*_ST_* value using SNP loci (F*_ST_* = 0.01667, *p* < 0.05). The pairwise F*_ST_* values between the two populations ranged from 0.01356 to 0.01678 (Figure 5), and the genetic differentiation between the two populations reached a significant level (*p*  <  0.05). The highest pairwise F*_ST_* value (0.01678) was observed between the DL and QD populations, while the lowest value (0.01356) was found for the DL and YT populations. The pairwise F*_ST_* value between the YT and QD populations was 0.01360. The results indicate that the pairwise F*_ST_* values in all three pairwise populations showed low levels of genetic differentiation (F*_ST_* < 0.05). 

**Figure 6:**
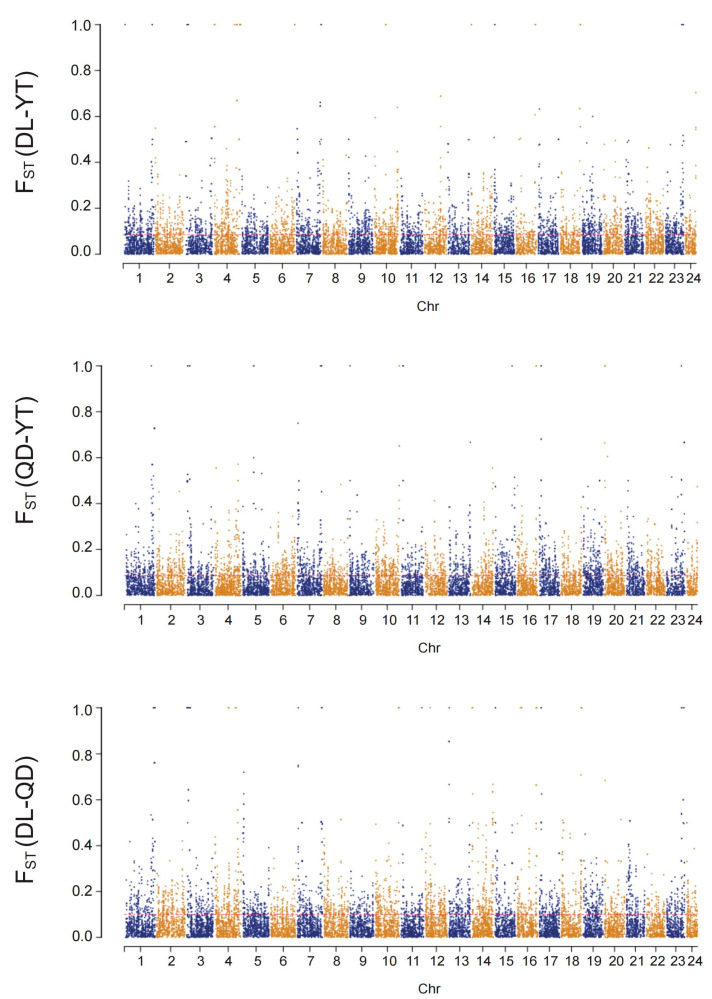
Population divergence (F*_ST_*) between the QD, DL, and YT populations. Mean F*_ST_* values are plotted in sliding windows of 100 kb with a 10 kb step size.

In addition, the number of loci at high levels of differentiation (F*_ST_* ≥ 0.15) in F_ST_ values for pairwise populations (between DL and QD, between DL and YT, and between QD and YT) were 1770, 1325, and 1374, respectively. Comparatively, the number of loci at moderate differentiation levels (0 < F*_ST_* < 0.15) for pairwise populations were 11,743, 10,839, and 11,115, respectively (Figure 6).

### 3.4. Genome-Wide Selection Pressure Analysis

**Figure 7:**
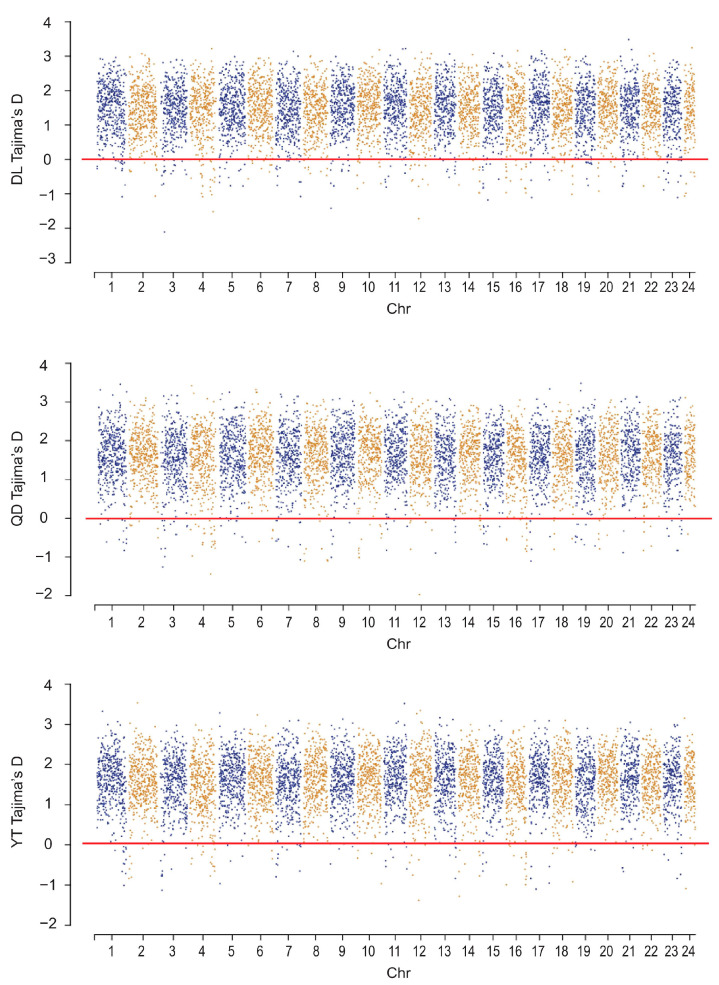
Distribution of selective sweep regions in *S. schlegelii* genome.

To compare the signatures of selective sweeps among the three *S. schlegelii* populations, a total of 225, 158, and 118 regions were analyzed under purifying selection (Tajima’s D < 0) based on a global database (149,434 SNPs) for DL, QD, and YT populations, respectively (Figure 7). These selectively swept regions were evenly distributed in 24 chromosomes of *S. schlegelii*, with a number of them showing high levels of selective sweeps (Figure 7). 

**Figure 8:**
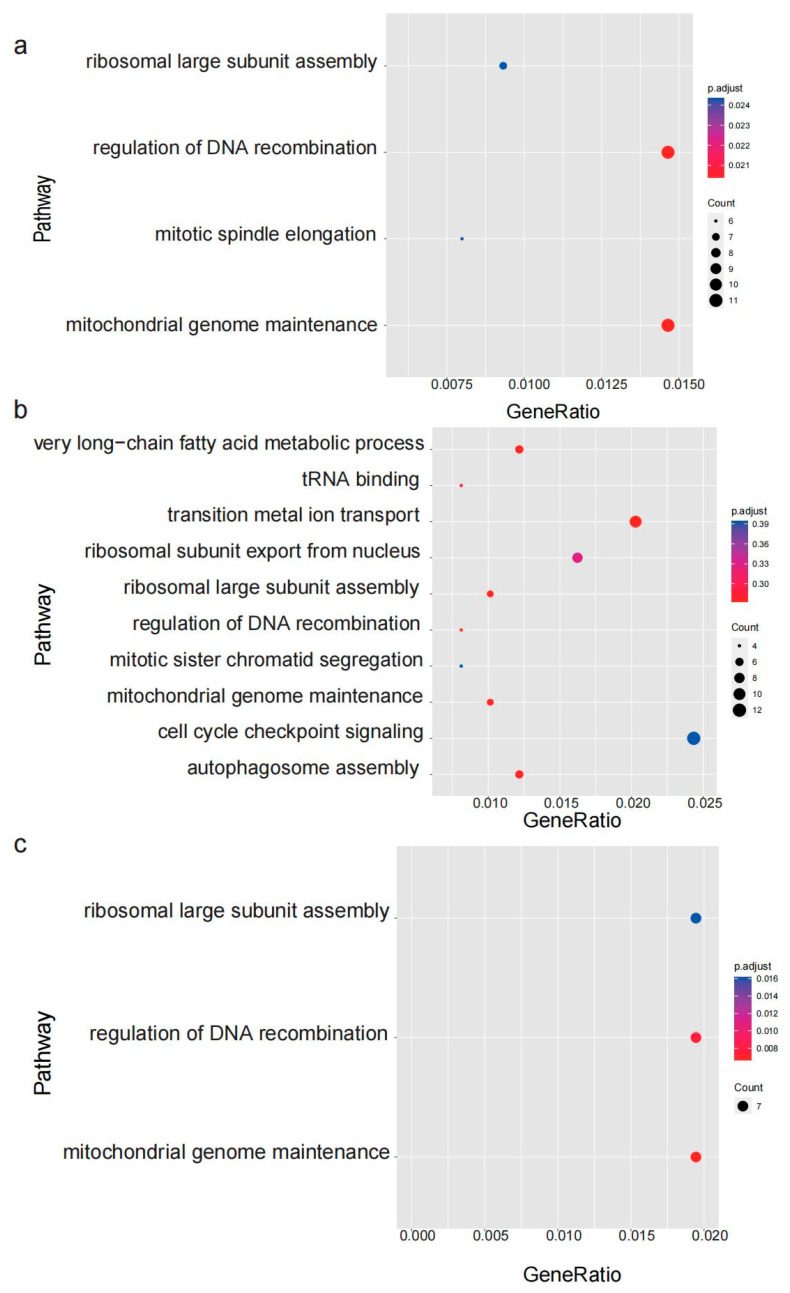
GO enrichment analysis of genes under selective sweeps in three population of *S. schlegelii*. (**a**) DL population; (**b**) QD population; and (**c**) YT population.

These swept genomic regions overlapped with 774 protein-coding genes in DL, 506 genes in QD, and 370 genes in YT populations. Gene ontology functional enrichment analysis of the swept genes also revealed that the number of 15, 33, and 7 genes, respectively, in the DL, QD, and YT populations were involved in important functions (Figure 8). Interestingly, none of the swept genes were shared with each other among the three populations, yet these genes were enriched in the same pathways such as the regulation of DNA recombination, ribosomal large subunit assembly, and mitochondrial genome maintenance. In addition, the function of swept genes among the different populations also showed differences. For instance, some swept genes in the DL population were enriched in mitotic spindle elongation associated with sexual maturity, which may contribute to the breeding of superior varieties. In the QD population, some genes were involved in very long-chain fatty acid metabolic processes associated with energy metabolism, which may provide a theoretical foundation for future research on the conservation and sustainable utilization of *S. schlegelii* resources.

## 4. Discussion

### 4.1. Genetic Differentiation Analysis

*S. schlegelii,* as an important economic fish species, is widely distributed in the northern sea area of China [22]. Previous studies have revealed that no significant morphological variations and no obvious genetic differentiation were detected among the above three *S. schlegelii* populations (DL, QD, and YT) based on analyses of microsatellite DNA loci [8] and the mitochondrial DNA control region [16]. In this study, we investigated the genetic differentiation F*_ST_* of *S. schlegelii* populations in the northwestern Pacific using GBS-based SNP data belonging to genome-wide markers which achieved deeper coverage than previous markers. F*_ST_* values between 0.15 and 0.25 indicate high differentiation, F*_ST_* values between 0.05 and 0.15 indicate moderate differentiation and F*_ST_* < 0.05 indicate no differentiation [35]. Analysis of genetic differentiation among above three populations based on Global F*_ST_* revealed low genetic differentiation. In addition, all of the pairwise F*_ST_* values between the populations indicated low levels of genetic divergence (F*_ST_* < 0.05), consistent with the previously reported lack of population genetic differentiation of *S. schlegelii* based on mtDNA control region sequences [16,17]. This result was also in accordance with the results of Peng et al., who used comparative mtDNA loop analysis to study the population structure and genetic diversity of the *S. schlegelii* populations in the Bohai Sea (Qingdao, Jinzhou, and Dalian), where there was no significant population genetic structural differences and low population genetic differentiation (F*_ST_* = −0.0113–0.0061) within the geographical range of the *S. schlegelii* population [36]. In addition, the pairwise F*_ST_* values obtained were also similar to those obtained by Cao et al. (wild populations: F*_ST_* < 0.05) with a similar sampling design and 2b-RAD sequencing method [37]. In addition, the pairwise F*_ST_* level of *S. schlegelii* populations (F*_ST_* = 0.01356, 0.01360, and 0.01678) was less than many other marine organisms, such as *Clupea pallasii* (F*_ST_* = 0.064–0.106) [38], *Acanthopagrus latus* (the highest F*_ST_* was 0.4288) [39], and *Brachionichthys hirsutus* (overall F*_ST_* = 0.043) [40]. This could confirm that *S. schlegelii* populations showed relatively low levels of genetic divergence among these two areas (QD, YT, and DL). 

We hypothesized that the low levels of genetic differentiation among geographic populations might be due to two factors. First, it may be attributable to the swimming capability of *S. schlegelii* larvae. They live on the surface of the ocean before the adult stage and can disperse along drifting seaweeds and eat plankton with high dispersal potential, which leads to extensive gene flow between populations of *S. schlegelii* in the northwestern Pacific [18]. Second, during the main spawning seasons (April to June each year) of *S. schlegelii,* the Yellow Sea Warm Current is characterized by the influx of relatively warmer water from the East China Sea into the Yellow Sea; the China Coastal Current, flowing southward along the eastern coast of China, transports water and materials, interacting with the Yellow Sea Warm Current [41]. Sea currents can carry plankton and a large number of *S. schlegelii* larvae to further areas, which suggests that the movement of *S. schlegelii* occurs frequently between geographic populations [42]. However, this hypothesis needs to be tested with future studies.

### 4.2. Genetic Diversity and Selection Pressure Analysis

The role of genetic diversity in the world is related to the survival, reproduction, fitness, and evolution of species [43]. Parameters such as *Ho*, *He*, *PIC*, and *π* based on SNPs are often used as criteria to evaluate population genetic diversity [44]. In our study, the *Ho* (0.14316–0.17684), *He* (0.14035–0.17145), and *PIC* (0.20672–0.24678) of *S. schlegelii* populations (DL, QD, and YT) showed an obviously lower level compared to the results (*Ho* = 0.2220–0.2271, *He* = 0.2280–0.2297, and *PIC* = 0.989–0.911) of *Pampus echinogaster* near the QD and DL coastal waters of China based on SNPs markers in a previous study [45]. This finding indicated that the number of loci possibly play a key role in genetic diversity. Analyses of *π* (7.63 × 10^−6^–8.77 × 10^−6^) within all three populations of *S. schlegelii* revealed generally lower levels than many other marine organisms, such as *Apostichopus Japonicas* in Yantai (7.92 × 10^−3^) [46], *Harpadon nehereus* (0.68833) [47], and *Sebastiscus marmoratus* (0.136) [48]. The above outcomes demonstrate that all three *S. schlegelii* populations with low levels of genetic diversity have poor capabilities to adapt to changing environmental conditions. Compared with our results, the slight difference in genetic diversity levels among the three populations might be largely caused by gene flow, which is consistent with the above genetic differentiation level. 

However, the structure of *S. schlegelii* showed no obvious genetic differentiation among different geographical populations. The high level of genetic divergence (pairwise F*_ST_* values > 0.15) between populations indicates obvious genetic differentiation among different populations at outlier loci, which may be due to ecological adaptation or environmental pressures. A previous study demonstrated that negative effects of climate change on the distribution patterns of *S. schlegelii* would result in varying degrees of habitat reduction [49]. In our study, the selective swept genes among the populations may have differed, but their main functions were all oriented toward biological processes, such as DNA recombination, ribosomal large subunit assembly, and mitochondrial genome maintenance, which may contribute to their environmental adaptability [50,51,52,53]. Unique swept genes were discovered in each population. For instance, in the DL population, the C-reactive protein gene (*Ssc*_10008587) enriched in the mitotic spindle elongation term may promote host defense via agglutination, phagocytosis, and complement fixation with calcium-dependent phosphorylcholine binding [54]; in the QD population, the gene (*Ssc*_10015638) could be mediated by G proteins which activate adenylyl cyclase and are involved in very long-chain fatty acid metabolic processes associated with energy metabolism. 

### 4.3. Population Structure and Environmental Adaptation

The PCA plot displayed a mixed distribution of individuals, indicating that they could not be clearly separated by three eigenvectors, which further supported the phenomenon that there is no significant genetic difference among individuals from different geographic populations. These results were also consistent with the topology of the phylogenetic tree and the ADMIXTURE plot. Furthermore, no obvious geographical barrier existed among different sampling locations. Therefore, the free-floating eggs and larvae could be easily transported by strong coastal currents during the breeding season. The frequent gene flow and drift caused a low level of genetic differentiation among populations along the coast of China. Our results may provide a theoretical foundation for future environmental changes and for implementing adaptive management measures of *S. schlegelii* resources. 

## 5. Conclusions

In conclusion, our study demonstrated that *S. schlegelii* populations showed low levels of genetic differentiation and genetic diversity among three populations of QD, DL, and YT in the Bohai and Yellow Seas. This phenomenon can guide the selection of populations with distant genetic relationships for propagation and release, which can promote genetic exchange within the population, increase the genetic diversity of the gene pool, and thereby enhance the adaptability and survival capacity of the population. Using selection pressure analysis, we found that the selective swept genes were mostly enriched in some biological processes involved in environmental adaptation. These findings help provide a theoretical basis for further studies aimed at protecting and utilizing *S. schlegelii* resources.

## Figures and Tables

**Table 1:** A summary of the SNPs and indels identified in the three populations of *S. schlegelii*.

		DL	QD	YT
Samples		20	20	20
SNPs		157,778	174,480	188,756
Indels		42,033	48,195	50,396
Intergenic		107,709 (68.27%)	120,457 (69.04%)	129,432 (68.57%)
Intron		88,180	98,059	105,455
Exon		4444	4810	4948
	Synonymous	2377 (53.49%)	2690 (55.93%)	2662 (53.80%)
	Non-synonymous	2067 (46.51%)	2120 (44.07%)	2316 (46.20%)
Splicing		628	644	703
Upstream		31,285	36,050	38,776
Downstream		31,507	35,416	37,971

**Table 2:** Population genetic diversity parameters of *S. schlegelii*.

Population	Observed Heterozygosity (*Ho*)	Expected Heterozygosity (*He*)	Polymorphism Information Content (*PIC*)
DL	0.14316	0.14035	0.20672
QD	0.15809	0.15420	0.22616
YT	0.17684	0.17145	0.24678

## Data Availability

Sixty GBS genome-wide raw data produced by Illumina HiseqTM2500 have been deposited in the Genome Sequence Archive (SRA) database (https://ngdc.cncb.ac.cn/gsa/browse/CRA008609) (accessed on 10 February 2023) under the accession number CRA008609.

## Supplementary Materials

The following supporting information can be downloaded at: https://www.mdpi.com/article/10.3390/genes15050534/s1, Table S1. The number of SNPs per filtering step.
