# Supplementary material for: Population Genomics of Commercial Fish Sebastes schlegelii of the Bohai and Yellow Seas (China) Using a Large SNP Panel from GBS
Source: Genes (Basel). 2024 Apr 24;15(5):534. doi: 10.3390/genes15050534 (PMC11121270; doi:10.3390/genes15050534)
Supplement: Supplementary file 1 [file genes-15-00534-s001.zip › genes-2944590-supplementary.pdf]

Table S1. The number of SNPs per filtering step

Filtering steps	The numbers of SNPs
SNPs presented in both two pipelines among three populations	157,167
SNPs after filtering step2	157,082
SNPs after filtering step3	156,864
SNPs after filtering step4	156,863
SNPs after filtering step5	153,154
SNPs after filtering step6	149,434
